# Development of a *Drosophila *S2 insect-cell based placental malaria vaccine production process

**DOI:** 10.1186/1753-6561-7-S6-P20

**Published:** 2013-12-04

**Authors:** Wian A de Jongh, Mafalda dos SM Resende, Carsten Leisted, Anette Strøbæk, Besim Berisha, Morten A Nielsen, Ali Salanti, Kathryn Hjerrild, Simon Draper, Charlotte Dyring

**Affiliations:** 1 ExpreS2ion Biotechnologies, Horsholm 2970, Denmark; 2 Centre for Medical Parasitology, Copenhagen University, Copenhagen 1356, Denmark; 3 The Jenner Institute, University of Oxford, Oxford, OX3 7DQ, UK

## Background

Malaria during pregnancy is the cause of 1500 neonatal deaths a day. Moreover, 40% of all low weight births are caused by pregnancy associated malaria. Researchers at Copenhagen University have identified the VAR2CSA protein as a potential protective recombinant placental malaria vaccine. ExpreS^2^ion Biotechnologies is responsible establishment of cell lines expressing VAR2CSA variants and for developing the protein production process based on VAR2CSA.

The ExpreS^2 ^System is a one-for-all protein expression system based on *Drosophila *S2 cells that is excellent in all phases of Drug Discovery, R&D and manufacturing due to high-level transient transfections, easy establishment of stable polyclonal pools that provides continuous high protein expression levels without selection pressure, and simple cloning procedure. It is a novel, non-viral, insect-cell based expression technology applied to the development of a critically needed vaccine. The VAR2CSA protein, which the vaccine is based on, is hard to express and comparison studies between insect, bacteria and yeast have shown that an insect cell system is the only one leading to a clinically useful immune response. Process optimization is also critically important, as the cost of manufacture must be as low as possible to allow the vaccine to be used in the countries where it is most needed.

## Aim

The choice and cost of a manufacturing platform is one of the most important strategic decisions in recombinant subunit vaccine development. Furthermore, the geographic distribution of malaria and the philanthropic funding sources involved requires production to be as cost-effective as possible. Single-use provides manufacturing flexibility and economic advantages, both highly desirable in this type of process. We therefore aim to develop cost-effective *Drosophila *S2 based Placental and Blood-stage malaria vaccine production processes combining the ExpreS^2 ^constitutive insect cell expression system with single-use bioreactor technology.

## Materials and methods

Thirty-four truncation variants of the ***VAR2CSA ***placental malaria vaccine antigen and full-length *Pf**Rh5 ***were cloned into pExpreS^2 ^vectors and transfected into *Drosophila *S2 insect cells. Stable cell lines were established in three weeks in T-flask culture, which were then inoculated at 8E6 cells/ml in shake flasks, or batch or fed-batch production in DasGip Bioreactors and harvested after 3 and 7 days respectively. The cultures were harvested by centrifugation and filtration, where after the proteins were purified using Ni^++ ^affinity columns and gel filtration. Bioreactor optimisation were performed in 1L DasGip mini-bioreactors, 2L Braun glass bioreactor, and the single-use CellReady3L bioreactor. Alternating Tangential Flow (ATF) technology from Refine was also employed for perfusion production tests. The bioreactor conditions were 25°C, pH6.5, Dissolved Oxygen 20%, 110 rpm stirrer speed using a Marine impeller. The perfusion rate was set to 0.5 to 3 Reactor Volumes (RV) per day, but was increased significantly faster for the CellReady 3L perfusion run compared to the Braun runs, with 3 RV per day reached by day 6 vs. day 9 for the Braun runs.

## Results

Thirty-four protein variants of VAR2CSA were screened for expression level. Further process optimization was performed on the lead candidate in glass bioreactors, and >30% yield increase was achieved using a fed-batch approach (results not shown). The expression of Rh5 was compared in batch, fed-batch and perfusion using both CellReady3L and glass bioreactors. There was no significant difference between growth in the DasGip bioreactor and the disposable CellReady bioreactor.

**Figure 1:**
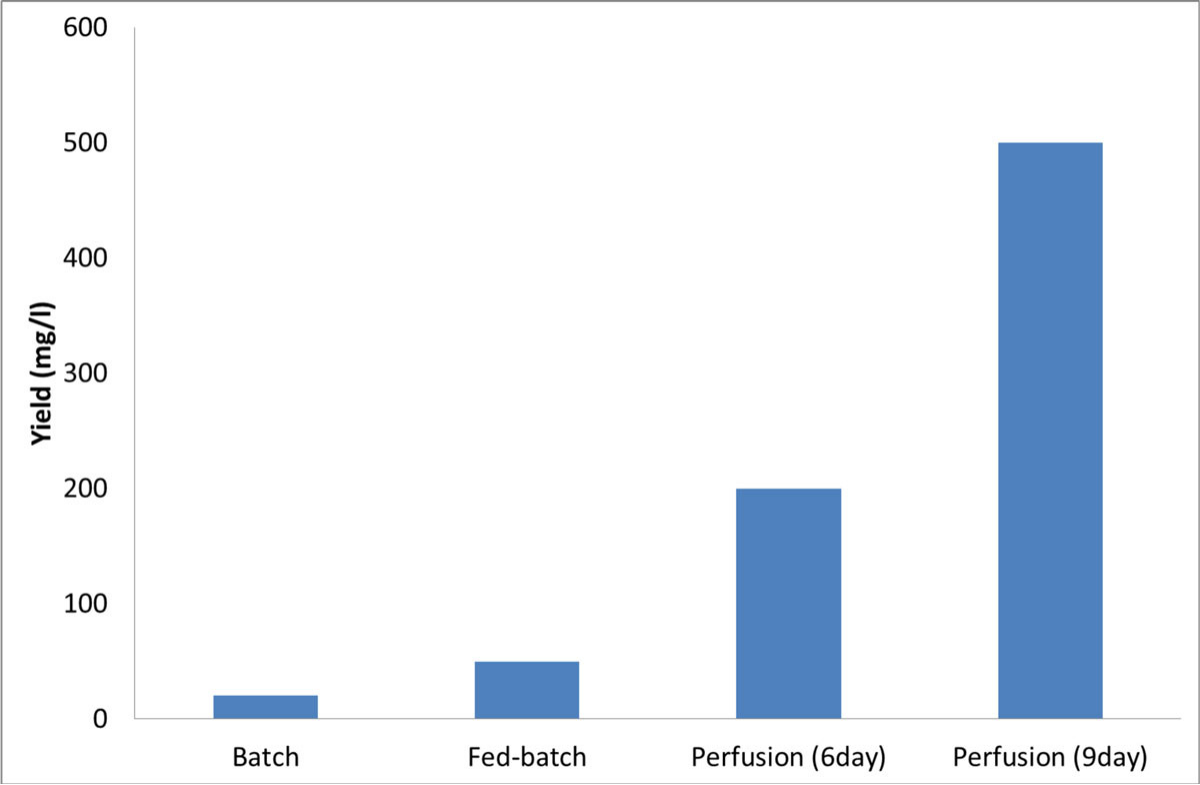
**Expression yields obtained for Rh5 in batch, fed-batch and ATF-perfusion modes**. Using perfusion could significantly increase yields.

Comparable yields were obtained in both systems whether running in batch, fed-batch, or perfusion mode (e.g. Perfusion day 6: 190 vs. 210 mg/L, results not shown). Furthermore, **350E6 cells/ml **were achieved in concentrated perfusion mode using the ATF and CellReady3L. Concentrated perfusion lead to final Rh5 yields of 210 mg/L and **500 mg/L **after 6 or 9 days production runs (see Figure 1).

## Conclusions

The ExpreS^2 ^platform has demonstrated its robustness of expression ability, by expression of two complex malaria antigens; and in breadth of hardware adaptability, as it was shown to perform comparably in the single use CellReady3L and glass bioreactors. Furthermore, extremely high cell counts and yields of Rh5 were achieved in Fed-batch and perfusion modes. The results demonstrate how the ExpreS^2 ^expression system in conjunction with single-use technology can be used to produce cost-effective malaria vaccines.

